# Screening for Q Fever in Patients Undergoing Transcatheter Aortic Valve Implantation, Israel, June 2018–May 2020

**DOI:** 10.3201/eid2708.204963

**Published:** 2021-08

**Authors:** Nesrin Ghanem-Zoubi, Mical Paul, Moran Szwarcwort, Yoram Agmon, Arthur Kerner

**Affiliations:** Rambam Health Care Campus, Haifa, Israel (N. Ghanem-Zoubi, M. Paul, M. Szwarcwort, Y. Agmon, A. Kerner);; Technion–Israel Institute of Technology, Haifa (N. Ghanem-Zoubi, M. Paul, Y. Agmon, A. Kerner)

**Keywords:** Q fever, *Coxiella burnettii*, infective endocarditis, transcatheter aortic valve implantation, bacteria, Israel

## Abstract

Q fever infective endocarditis frequently mimics degenerative valvular disease. We tested for *Coxiella burnettii* antibodies in 155 patients in Israel who underwent transcatheter aortic valve implantation. Q fever infective endocarditis was diagnosed and treated in 4 (2.6%) patients; follow-up at a median 12 months after valve implantation indicated preserved prosthetic valvular function.

Q fever is a zoonotic infection caused by the bacterium *Coxiella burnettii* that occurs worldwide. Q fever has been endemic in Israel for many years; several superimposed outbreaks have occurred in the past 2 decades (*1*–*3*).

A clinical observation of 2 patients with severe prosthetic Q fever infective endocarditis (IE) diagnosed several months after transcatheter aortic valve implantation (TAVI) indicated that Q fever IE could have been the underlying valve disease but was not detected before TAVI. We considered this possibility, because Q fever IE typically manifests as a chronic disease, frequently in the absence of fever and inflammatory markers, as well as absent or small fine vegetations (*4*,*5*).

Considering the epidemiology of Q fever in Israel and the ominous prognosis of Q fever endocarditis after TAVI, we began routine screening of patients undergoing TAVI for antibodies to *C. burnettii* to identify and treat Q fever IE as soon as possible after TAVI. In this study, we review a 2-year period of serologic screening and discuss the value of Q fever screening in this setting.

## The Study

Beginning in June 2018, serologic screening for Q fever was ordered for all patients admitted for TAVI at Rambam Health Care Campus, a 960-bed primary and tertiary university-affiliated hospital in northern Israel. We tested serum samples for *C. burnetii* phase 2 IgM and phase I or phase II IgG by using ELISA (Institute Virion/Serion GmbH, https://www.virion-serion.de). For samples that tested positive, we then conducted an indirect immunofluorescence assay (IFA) for confirmation and titer determination. We performed the IFA locally using a commercial kit (Focus Diagnostics, https://www.focusdx.com) or an in-house test at The National Reference Laboratory for Rickettsiosis (Nes Ziona, Israel). An infectious diseases specialist evaluated patients with positive IgG for *C. burnettii* chronic infection. IE was diagnosed according to the modified Duke criteria (*6*) or the Dutch consensus guidelines of chronic Q fever infection (*7*) with an IFA phase I IgG of >800. Patients began treatment and follow-up was conducted at the infectious disease and cardiology outpatient clinics. Diagnostic testing was performed as a part of a clinical routine, and anonymous data collection was approved by the hospital's ethics committee with a waiver of informed consent.

During June 1, 2018–May 31, 2020, a total of 197 TAVI procedures were performed at Rambam Health Care Campus. Serologic testing for Q fever was conducted in 155 patients. Nine patients tested positive for >1 Q fever IgG by ELISA: 7 had phase I IgG and 2 patients had only phase II IgG. On IFA, 4 patients (2.6%) had a phase I IgG titer of >800 and were further evaluated for Q fever IE (Table). All 4 patients had underlying conditions, but none had fever or vegetations on echocardiography. None of the patients had a specific high-risk exposure for Q fever. We recommended treatment with doxycycline and hydroxychloroquine for >24 months (as recommended for Q fever IE in the presence of prosthetic valve). In 3 of 4 patients, treatment was modified to an alternative regimen because of intolerance or side effects. We did not perform fluorodeoxyglucose positron emission tomography-computed tomography for diagnosis, because it would not have led to a change in management. Patient 2 underwent fluorodeoxyglucose positron emission tomography–computed tomography 2 months before TAVI as part of lymphoma follow-up; it showed no evidence of pathologic uptake in the valve or elsewhere. As of the last follow-up visit (median 12 months, range 8–18 months), all 4 patients had preserved prosthetic valve function, and none experienced symptomatic Q fever infection. One patient reported severe fatigue, likely related to underlying scleroderma.

**Table:** Characteristics of identified patients with Q fever infective endocarditis, Israel, June 1, 2018–May 31, 2020*

Variable	Patient 1	Patient 2	Patient 3	Patient 4
Age, y/sex	77/M	52/F	73/F	79/M
Underlying conditions	Hypertension, CAD, s/p CABG, and AVR (7 y)	s/p Hodgkin lymphoma (30 y), DM, CAD, and s/p CABG (8 y)	Scleroderma	DM, hypertension, asthma
Habitat/exposure risk factor†	Urban/none	Urban/none	Urban/none	Urban/none
Indication for TAVI	Symptomatic aortic insufficiency, NYHA 3/4	Symptomatic aortic stenosis and insufficiency (moderate to severe); chest pain and dyspnea with minimal effort	Symptomatic severe aortic stenosis; recurrent syncope	Symptomatic severe aortic stenosis, NYHA 3/4
Echo findings before TAVI	Moderate aortic stenosis and severe regurgitation with thickened leaflets	Severe aortic stenosis	Severe aortic stenosis with severe calcifications and moderate mitral regurgitation with leaflets sclerosis	Severe aortic stenosis
*Coxiella burnettii* phase I IgG	1:32,00	1:25,600	1:3,200	1:1,024
*C. burnettii* PCR in blood	Not performed	Not performed	Negative	Not performed
Q fever IE according to modified Duke criteria	Possible	Possible	Possible	Possible
Q fever IE according to Dutch consensus guidelines	Probable	Probable	Probable	Probable
Treatment	Doxycycline and hydroxychloroquine, changed to doxycycline and ciprofloxacin	Doxycycline and hydroxychloroquine, changed to doxycyline monotherapy	Doxycycline and hydroxychloroquine, changed to ciprofloxacin	Doxycycline and hydroxychloroquine
Timing of and status at last follow-up	18 mo, asymptomatic, preserved valve function, and stable serologic results	8 mo, asymptomatic, preserved valve function, and stable serologic results	12 mo, severe fatigue, preserved aortic valve function, and stable serologic results	12 mo, asymptomatic, preserved valve function, and decreasing serologic results

*AVR, aortic valve replacement; CABG, coronary artery bypass grafting; CAD, coronary artery disease; DM, diabetes mellitus; IE, infective endocarditis; NYHA, New York Heart Association (classification); s/p, status post; TAVI, transcatheter aortic valve implantation. †Risk factors for Q fever are employment as a veterinarian, farmer, abattoir worker, or any contact with farm animals.

## Conclusions

During a 2-year period of routine serologic screening for Q fever among patients undergoing TAVI, we identified 4 case-patients with Q fever IE, affecting 2.6% of patients screened. None of the 4 case-patients experienced fever or echocardiographic findings that were suggestive of IE.

Diagnosing Q fever IE can be challenging, especially in the absence of tissue samples, as in the case of patients undergoing TAVI. Several studies have highlighted the difficulties of the diagnosis of Q fever IE (Appendix Table 1). The diagnostic criteria used in the absence of tissue samples are based on the modified Duke criteria (*6*), the Dutch consensus guidelines for chronic Q fever (*7*), and the recently revised definition of “persistent *C. burnettii* infection” by Melenotte et al. (*8*) (Appendix Table 2). For definitive diagnosis, all 3 definitions are based mainly on serologic tests and echocardiography, PET, or CT findings to prove valve infection. Both imaging modalities have poor sensitivity in the case of *C. burnettii* IE (*9*–*11*). The alternative minor diagnostic criteria consist also of infrequent findings, such as embolic and immunologic phenomena. We recommended treatment for patients with possible or probable IE (Table), recognizing the significant consequences of a delayed diagnosis and treatment of prosthetic Q fever IE among patients at very high risk for surgery a priori.

In a study conducted in 2 centers in the United Kingdom, routine serologic screening for Q fever before valve surgery was performed in 139 patients. In this low-endemicity setting, no patient with Q fever IE was identified (*12*). In our study conducted in a Q fever–endemic region, the yield of such a strategy seems clinically significant. The incidence of Q fever in Israel according to reported cases to the Ministry of Health is ≈2.2/100,000 population (https://www.health.gov.il/UnitsOffice/HD/PH/epidemiology/Pages/epidemiology_report.aspx). In comparison, data from countries in the European Union from 2018 showed the highest incidence was 0.7/100,000 population in Spain. An alternative indicator of Q fever endemicity is the percentage of IE caused by Q fever out of all IE cases. According to the International Collaboration on Endocarditis registry data, *C. burnettii* was responsible for almost 1% of all IE cases in 25 countries (*13*). This rate reaches almost 5% in Q fever–endemic regions, such as southern France (*14*). A similar rate was observed at our hospital; Q fever IE was diagnosed in 5 (5.3%) of 95 cases of definitive IE during 2013–2016, according to local data from a prospective registry.

The primary limitation of our study is that, as a single-center study, it reflects the epidemiology of a limited geographic area. The short-term follow-up of patients with Q fever IE does not enable a description of the long-term benefit of our strategy. We did not evaluate the cost-effectiveness of our surveillance strategy. In addition, we might have missed cases of Q fever IE by conducting serologic screening only, since Q fever IE with low phase I IgG titers (<800) (*9*) or even negative serologic results (*15*) has been described. Nevertheless, as a screening strategy, serologic testing seems to be sufficient. Early diagnosis and appropriate treatment as soon as possible after prosthetic valve implantation contributed substantially to preserving valve function and prevented potential ongoing infection. Therefore, we suggest screening for Q fever in TAVI patients in settings in which Q fever incidence is >0.5 per 100,000 (nationally or in Q fever–endemic regions within countries), after Q fever outbreaks regardless of baseline incidence, or in places in which Q fever causes >2% of all cases of IE.

Appendix Additional information on screening for Q fever in patients undergoing transcatheter aortic valve implantation, Israel, June 2018–May 2020.
