# Supplementary material for: Screening for Q Fever in Patients Undergoing Transcatheter Aortic Valve Implantation, Israel, June 2018–May 2020
Source: Emerg Infect Dis. 2021 Aug;27(8):2205–7. doi: 10.3201/eid2708.204963 (PMC8314821; doi:10.3201/eid2708.204963)
Supplement: Appendix — Additional information on screening for Q fever in patients undergoing transcatheter aortic valve implantation, Israel, June 2018–May 2020. [file 20-4963-Techapp-s1.pdf]

Article DOI: https://doi.org/10.3201/eid2708.204963

Screening for Q Fever in Patients Undergoing Transcatheter Aortic Valve Implantation, Israel, June 2018–May 2020

Appendix

Appendix Table 1. Studies describing the "accidental" diagnosis of Q fever infective endocarditis

Reference	Study design, country	Study population	Main results
Kampschreur et al. (*1*)	Case report, the Netherlands		Description of 3 patients with delayed diagnosis of Q fever IE until after valve surgery
Salamand et al. (*2*)	Case series, 14 y, single institution, France	Description of 19 patients with Q fever IE who underwent surgical intervention	8 of 19 patients with Q fever IE who underwent surgical intervention and had a late diagnosis, either during or after surgery
Grisoli et al. (*3*)	Cohort study, 14 y, single institution, France	All resected cardiac valves or prostheses underwent routine histologic examination, on a microbiologic and molecular biologic basis, in addition to serologic testing for fastidious microorganisms.	14 (0.2%) of 6,401 examined valves were diagnosed with "unsuspected" Q fever IE.
Shapira et al. (*4*)	Cohort study, 10 y, single center, Israel	All excised valves were cultured and underwent histologic examination for the presence of inflammatory infiltrates, vegetations, and microorganisms. Patients with findings suggestive of inflammation underwent serologic investigation.	1 of 8 patients with histologic endocarditis (of 857 examined valves) received a diagnosis of Q fever IE.
Wiener et al. (*5*)	Case series, 9 y, single center, Israel	The clinical and serologic manifestations of 9 patients who received a diagnosis of Q fever IE were reviewed.	3 out of 4 operated cases were diagnosed only following surgery

Appendix Table 2. Q fever infective endocarditis definitions in the absence of tissue samples*

Modified Duke criteria (*6*)	Dutch consensus guidelines (*7*)	French NRC definition (*8*)
1.Positive blood culture for *Coxiella burnetii* or anti-phase 1 IgG titer >1:800	1. IFA ≥1:800 or 1:1,024 for *C. burnetii* phase I IgG	1. Positive culture or PCR of the blood or emboli or serologic tests with IgG phase I ≥6400
2. Echocardiographic findings of IE, such as vegetations, abscesses, etc.†	2. Modified Duke criteria<br>3. Valvular infection proven by FDG PET-CT‡	2. Echocardiographic findings of IE-vegetations, abscesses, etc. or PET scan displaying a specific valve fixation and mycotic aneurysm†
3. Minor criteria: a) Predisposing heart disease; b) Fever >38°C§; c) Vascular phenomena¶; d) Immunologic phenomena#	4. Valvulopathy including prosthetic valve not meeting the major criteria of the modified Duke criteria	4. Minor criteria: a) Predisposing heart condition; b) Fever >38°C§; c) Vascular phenomena¶; d) Immunologic phenomena#; e) IgG1 antibody titers ≥800 and <6400
Endocarditis definitions<br>Definite IE: 1+2 or 1+≥3 minor criteria; possible IE: 1+≥1 minor criteria	Proven IE: 1+2 or 1+3; probable IE: 1+4	Definite IE: 1+2 or 2+3 minor criteria including a+e or 1+3 minor criteria including a; Possible IE: 1+2 minor criteria or 2+2 minor criteria or 3 minor criteria**

*FDG PET-CT, fluorodeoxyglucose positron emission tomography-computed tomography; IE, infective endocarditis; IFA, immunofluorescence assay; NRC, National Reference Center.
†Absent in >50% of cases (*9*).
‡Positive in 13%–20% of cases (*10*–*12*).
§Absent in 20%–40% of cases (*9*,*13*).
¶Exist in less than 20% of cases (*13*). Vascular phenomena include major arterial emboli, septic pulmonary infarcts, mycotic aneurysm, Intracranial hemorrhage, conjunctival hemorrhages, and Janeway lesions.
#Exist in less than 20% of cases (*13*). Immunologic phenomena include glomerulonephritis, Osler's nodes, Roth spots, or rheumatoid factor.
**Including 1 microbiologic characteristic and a cardiac predisposition.

References

1. Kampschreur LM, Hoornenborg E, Renders NHM, Oosterheert JJ, Haverman JF, Elsman P, et al. Delayed diagnosis of chronic Q fever and cardiac valve surgery. Emerg Infect Dis. 2013;19:768–70. PubMed https://doi.org/10.3201/eid1905.120353

2. Salamand AC, Collart F, Caus T, Casalta JP, Mouly-Bandini A, Monties JR, et al. Q fever endocarditis: over 14 years of surgical experience in a referral center for rickettsioses. J Heart Valve Dis. 2002;11:84–90. PubMed

3. Grisoli D, Million M, Edouard S, Thuny F, Lepidi H, Collart F, et al. Latent Q fever endocarditis in patients undergoing routine valve surgery. J Heart Valve Dis. 2014;23:735–43. PubMed

4. Shapira N, Merin O, Rosenmann E, Dzigivker I, Bitran D, Yinnon AM, et al. Latent infective endocarditis: epidemiology and clinical characteristics of patients with unsuspected endocarditis detected after elective valve replacement. Ann Thorac Surg. 2004;78:1623–9. PubMed https://doi.org/10.1016/j.athoracsur.2004.05.052

5. Wiener-Well Y, Fink D, Schlesinger Y, Raveh D, Rudensky B, Yinnon AM. Q fever endocarditis; not always expected. Clin Microbiol Infect. 2010;16:359–62. PubMed https://doi.org/10.1111/j.1469-0691.2009.02805.x

6. Li JS, Sexton DJ, Mick N, Nettles R, Fowler VG Jr, Ryan T, et al. Proposed modifications to the Duke criteria for the diagnosis of infective endocarditis. Clin Infect Dis. 2000;30:633–8. PubMed https://doi.org/10.1086/313753

7. Kampschreur LM, Wegdam-Blans MCA, Wever PC, Renders NHM, Delsing CE, Sprong T, et al.; Dutch Q Fever Consensus Group. Chronic Q fever diagnosis—consensus guideline versus expert opinion. Emerg Infect Dis. 2015;21:1183–8. PubMed https://doi.org/10.3201/eid2107.130955

8. Melenotte C, Protopopescu C, Million M, Edouard S, Carrieri MP, Eldin C, et al. Clinical features and complications of Coxiella burnetii infections from the French National Reference Center for Q fever. JAMA Netw Open. 2018;1:e181580. PubMed https://doi.org/10.1001/jamanetworkopen.2018.1580

9. Million M, Thuny F, Richet H, Raoult D. Long-term outcome of Q fever endocarditis: a 26-year personal survey. Lancet Infect Dis. 2010;10:527–35. PubMed https://doi.org/10.1016/S1473-3099(10)70135-3

10. Eldin C, Melenotte C, Million M, Cammilleri S, Sotto A, Elsendoorn A, et al. 18F-FDG PET/CT as a central tool in the shift from chronic Q fever to Coxiella burnetii persistent focalized infection: A consecutive case series. Medicine (Baltimore). 2016;95:e4287. PubMed https://doi.org/10.1097/MD.0000000000004287

11. Kouijzer IJE, Kampschreur LM, Wever PC, Hoekstra C, van Kasteren MEE, de Jager-Leclercq MGL, et al. The value of 18 F-FDG PET/CT in diagnosis and during follow-up in 273 patients with chronic Q fever. J Nucl Med. 2018;59:127–33. PubMed https://doi.org/10.2967/jnumed.117.192492

12. Melenotte C, Million M, Raoult D. New insights in *Coxiella burnetii* infection: diagnosis and therapeutic update. Expert Rev Anti Infect Ther. 2020;18:75–86. PubMed https://doi.org/10.1080/14787210.2020.1699055

13. Elzein FE, Alsherbeeni N, Alnajashi K, Alsufyani E, Akhtar MY, Albalawi R, et al. Ten-year experience of Q fever endocarditis in a tertiary cardiac center in Saudi Arabia. Int J Infect Dis. 2019;88:21–6. PubMed https://doi.org/10.1016/j.ijid.2019.07.035
